# Preclinical efficacy of the bioreductive alkylating agent RH1 against paediatric tumours

**DOI:** 10.1038/sj.bjc.6605100

**Published:** 2009-06-02

**Authors:** D Hussein, S V Holt, K E Brookes, T Klymenko, J K Adamski, A Hogg, E J Estlin, T Ward, C Dive, G W J Makin

**Affiliations:** 1 Clinical and Experimental Pharmacology Group, Paterson Institute for Cancer Research, Manchester, UK; 2 Department of Paediatric Oncology, Royal Manchester Children's Hospital, Manchester, UK; 3 School of Cancer and Imaging Sciences, Faculty of Medical and Human Sciences, University of Manchester, Manchester, UK

**Keywords:** childhood cancer, neuroblastoma, osteosarcoma, Ewing's sarcoma, novel therapies

## Abstract

**Background:**

Despite substantial improvements in childhood cancer survival, drug resistance remains problematic for several paediatric tumour types. The urgent need to access novel agents to treat drug-resistant disease should be expedited by pre-clinical evaluation of paediatric tumour models during the early stages of drug development in adult cancer patients.

**Methods/results:**

The novel cytotoxic RH1 (2,5-diaziridinyl-3-[hydroxymethyl]-6-methyl-1,4-benzoquinone) is activated by the obligate two-electron reductase DT-diaphorase (DTD, widely expressed in adult tumour cells) to a potent DNA interstrand cross-linker. In acute viability assays against neuroblastoma, osteosarcoma, and Ewing′s sarcoma cell lines RH1 IC_50_ values ranged from 1-200 nM and drug potency correlated both with DTD levels and drug-induced apoptosis. However, synergy between RH1 and cisplatin or doxorubicin was only seen in low DTD expressing cell lines. In clonogenic assays RH1 IC_50_ values ranged from 1.5–7.5 nM and drug potency did not correlate with DTD level. In A673 Ewing's sarcoma and 791T osteosarcoma tumour xenografts in mice RH1 induced apoptosis 24 h after a single bolus injection (0.4 mg/kg) and daily dosing for 5 days delayed tumour growth relative to control.

**Conclusion:**

The demonstration of RH1 efficacy against paediatric tumour cell lines, which was performed concurrently with the adult Phase 1 Trial, suggests that this agent may have clinical usefulness in childhood cancer.

Average 5–year survival rates for childhood cancers have improved dramatically over the past few decades, such that 75% of children with cancer can now expect to be cured (Gatta *et al*, 2005). However, cancer remains the second most common cause of death in children between the ages of 1 and 11 years. Although the overall survival for this group of patients is excellent, there remain selected tumour types in which survival is extremely poor, and for which acquired drug resistance is a major clinical problem. For example, for children over the age of 1 with stage 4 neuroblastoma, the best reported 3-year survival is only 55%, despite intensive chemotherapy, surgery and radiotherapy (Matthay *et al*, 1999). For those patients who fail to achieve adequate response to induction chemotherapy, survival can be expected to be very low (Katzenstein *et al*, 2004). Similarly, poor survival is seen in patients with metastatic Ewing's sarcoma and osteosarcoma, despite the vast majority of patients receiving intensive multi-agent chemotherapy in international collaborative trials. Thus, an urgent need exists for novel agents for this group of paediatric cancer patients.

Bioreductive agents are a promising area for anticancer drug discovery. These pro-drugs are converted to active intermediates by enzymatic activity either in hypoxic areas of solid tumours (Sartorelli, 1988) or by increased activity of these enzymes in tumour compared with normal tissues (Brown and Giaccia, 1998), and therefore should exhibit tumour-selective cytotoxicity. One- or two-electron reductases, including cytochrome p450 reductase (Bligh *et al*, 1990), NADH cytochrome b5 reductase (Hodnick and Sartorelli, 1993), and diphtheria toxin-diaphorase (DTD), also known as NAD(P)H:quinine oxidoreductase (NQ01) (Siegel *et al*, 1990; Gibson *et al*, 1992), can activate bioreductive drugs. The two-electron reductase DTD is particularly interesting, as its activity is elevated in a wide range of adult tumour types compared with normal tissue, and is not dependant upon tumour hypoxia (Cresteil and Jaiswal, 1991; Malkinson *et al*, 1992; Cummings *et al*, 1998). RH1 (2,5-diaziridinyl-3-[hydroxymethyl]-6-methyl-1,4-benzoquinone) was identified through its very high affinity for DTD (Beall *et al*, 1995; Winski *et al*, 1998). Reduction of RH1 facilitates the formation of GCC sequence-specific DNA interstrand cross-linked adducts (Berardini *et al*, 1993), and the induction of apoptosis within 6 h of exposure (Dehn *et al*, 2005). Diphtheria toxin-diaphorase activity and expression correlate with RH1-induced cytotoxicity in colon cancer and non-small-cell lung cancer cell lines *in vitro* (Winski *et al*, 1998; Sharp *et al*, 2000). In the NCI60 tumour cell line panel cell lines expressing NQ01 show marked sensitivity to RH1, but, in addition, a number of tumour types, particularly leukaemia and lymphoma cell lines, are sensitive to RH1 despite relatively low DTD activity (Tudor *et al*, 2005). RH1 is a substrate for other reductase enzymes, including cytochrome p450, although recent work suggests that this is unlikely to play a major role in its activation in cells with normal p450 activity (Begleiter *et al*, 2007), and that the one-electron reductase NRH:quinine oxidoreductase 2 (NQ02) may be responsible for the toxicity of RH1 in leukaemia and lymphoma cells (Yan *et al*, 2008). RH1 is active against human tumours in xenograft models in mice (Cummings *et al*, 2003; Dehn *et al*, 2004) where DNA cross-linking could be detected rapidly after RH1 dosing (Ward *et al*, 2005). Acquired tumour cell resistance to RH1 seems to be because of changes in drug uptake/efflux in line with other diazardinylbenzoquinone-resistant cell lines (T Ward, personal communication) (Ward *et al*, 1995).

RH1 has recently completed Phase 1 study in adults in the United Kingdom and further clinical evaluation is ongoing (Danson *et al*, 2007a, 2007b). The dose-limiting toxicity in this UK study was myelosuppression. To date, there has been no report on the efficacy of RH1 against paediatric tumour cell lines. Here we have investigated the anti-tumour activity of RH1 against neuroblastoma, Ewing's sarcoma, and osteosarcoma cell lines *in vitro* and against Ewing's sarcoma and osteosarcoma xenograft models.

## Materials and methods

### Paediatric tumour cell lines

The human neuroblastoma cell line pairs, SH-SY5Y and SH-EP1 and LA1-5S and LA1-55n, were kindly provided by Robert Ross (Fordham University, New York, NY, USA). RDES and A673 ESFT cells were kindly provided by Sue Burchill (CRUK Clinical Research Centre, St James's Hospital, Leeds, UK). The osteosarcoma cell lines 791T and U2OS were from the Paterson Institute cell bank. All cell lines were maintained in RPMI 1640 or DMEM F12 (Gibco, Paisley, UK) supplemented with 10% fetal bovine serum. Lysates of rhabdomyosarcoma cell lines were kindly provided by John Anderson (Institute of Child Health, London).

### Reagents and chemicals

RH1 was provided by Allos Therapeutics Inc. (Westminster, CO, USA), and dissolved in DMSO. Other cytotoxic anticancer drugs were purchased from Sigma-Aldrich (Gillingham, UK).

### Clonogenic assays

Clonogenic assays were performed as described earlier (Hussein *et al*, 2006). Cells were treated with varying concentrations of RH1 or with cytotoxic agent for 1 h, before the medium was aspirated, cells washed with PBS, and fresh medium added. Plates were incubated at 37°C and 5% CO_2_ for 7–10 days until visible colonies (>50 cells) were present in the untreated well. All assays were performed in triplicate. Surviving fraction was calculated as number of colonies in the test condition/number of colonies in the untreated well and plotted logarithmically against drug concentration.

### Growth assays

The sulforhodamine B (SRB) assay (Vichai and Kirtikara, 2006) was used to determine cell growth after exposure to RH1 and cytotoxic drugs. Cells were plated in exponential growth phase in 96-well plates at densities determined by initial growth curves and treated with varying concentrations of RH1 or cytotoxic agent for 1 h. Five days after drug treatment, when untreated cells were still in exponential growth phase, cells were fixed and stained before measuring absorbance at 540 nm using a microplate reader (Labsystems Multiskan EX, MTX Labsystems, Vienna, VA, USA). All assays were performed in triplicate and IC_50_ values were estimated using Calcusyn software (Biosoft, Cambridge, UK).

### Immunoblotting

Protein lysates were prepared as described earlier (Hussein *et al*, 2006) and resolved by SDS polyacrylamide gel electrophoresis and transferred to PVDF membrane (Immobilon, Millipore, Watford, UK). Standard immunoblotting procedures were followed with overnight incubation at 4 °C with the following primary antibodies: DT diaphorase 1 in 1000 (David Ross, University of Colorado), NQO2 1 in 1000 (David Siegel, University of Colorado), PARP 1 : 1000 (Cell Signalling, Beverly, MA, USA), cleaved caspase 3 1 : 100 (Cell Signalling), GAPDH 1 : 2000 (Abcam, Cambridge, UK), and actin 1 : 2500 (Sigma-Aldrich). Blots were visualised with the enhanced chemiluminescence system (GE Healthcare, Little Chalfont, UK) and analysed on a Fuji LAS-1000 Plus imaging system with AIDA software (Fuji, Bedford, UK).

### DCPIP assay of DTD activity

The DCPIP (2,6-dichlorophenolindophenol) assay was used to measure functional DTD levels in cell pellets of both the sensitive and resistant cell lines. Diphtheria toxin-diaphorase activity was determined by following the spectrophotometric dicoumarol inhibitable fraction of DCPIP reduction (Traver *et al*, 1992; Phillips *et al*, 1999). Pure recombinant human DTD (kindly supplied by David Siegel, University of Colorado) was used as a reference standard (Chen *et al*, 1992).

### Apoptosis assay

Cells were drug treated with RH1 for 1 h in exponential growth phase. After 24 h, cells were harvested and adherent and detached cells were combined. Cells were fixed in 1.2% formaldehyde/48% glycerol in TRIS/EDTA and nuclei stained with DAPI. The number of cells with nuclear apoptotic morphology was counted in five fields of 100 cells. Mean values were calculated from three independent experiments.

### Drug combination studies

Combination Index (CI) methodology was used to evaluate multiple drug-effect interactions with CalcuSyn software (Biosoft, Cambridge, UK) (Chou and Hayball, 1996). This uses the multiple drug-effect equation of Chou and Talalay (1977) derived from enzyme kinetic models. Additivity between two agents is denoted by a CI=0.9–1.1, with synergy and antagonism denoted by CI<0.9 and CI>1.1, respectively (Chou *et al*, 1994). This method for assessing synergy takes into account both the potency (*D*_m_ or inhibitory concentration 50% (IC_50_)) and shape of the concentration-effect curve. The ratio of RH1 to the cytotoxic agent of interest was fixed from the IC_50_ values from the SRB assay. Cells were treated with RH1 and cytotoxic for 1 h. Seven drug concentrations were used covering the concentration-effect curve. Linear correlation coefficients (*r*) were generated for each curve to determine the applicability of the data to the method of analysis. In all experiments, *r* was >0.9.

### Tumour xenografts

Balb/c-NUDE mice were used for all experiments. For A673 studies, 5 × 10^6^ A673 cells in 0.2 ml serum-free RPMI tissue culture media were implanted by a single subcutaneous (s.c.) injection into the right flank. For 791T studies, 3 × 10^6^ cells in a 50 : 50 mixture of serum-free RPMI tissue culture media and matrigel to a total volume of 0.2 ml were implanted s.c. in the right flank. Tumours were allowed to grow to 200 mm^3^, at which point animals were randomly assigned to control or treatment groups. RH1 treatment consisted of intraperitoneal (i.p.) injection of 0.4 mg kg^−1^ daily for 5 days as described earlier (Digby *et al*, 2005). This magnitude of dose has been shown to produce measurable DNA cross-linking in xenografted tumour tissue in mice (Ward *et al*, 2005). Control mice received 0.2 ml of 1% DMSO i.p. daily for 5 days. Mice were housed in an individually ventilated caging system on a 12-h light/dark environment maintained at constant temperature and humidity. The animals were fed a standard diet (Harlan-Teklad, Madison, WI, USA), and tumour volume (defined as length × width^2^ divided by 2) was measured daily. When tumour volume reached 1250 mm^3^, the mice were killed by a schedule 1 method and tumours were excised and formalin fixed and wax embedded before immunohistochemistry for cleaved caspase 3 (Cell Signalling). All procedures were carried out in accordance with UKCCCR guidelines 1999 by approved protocols (Home Office Project license no. 40-2804).

### Statistical analysis

Individual profile plots of tumour volume against days from randomisation were examined. After taking base 10 logarithms the plots were well approximated with straight lines. Formal fitting was then performed using linear mixed effect models, which incorporated animal-specific random intercepts and slopes. Primary focus lay in the difference in average slope between the RH1 and control groups for each tumour type separately. A pooled estimate and test of this primary parameter was obtained over the two tumour groups using meta-analysis methods given the estimated effect sizes and their standard errors.

## Results

### Diphtheria toxin-diaphorase expression and activity in paediatric tumour cell lines

**Figure 1:**
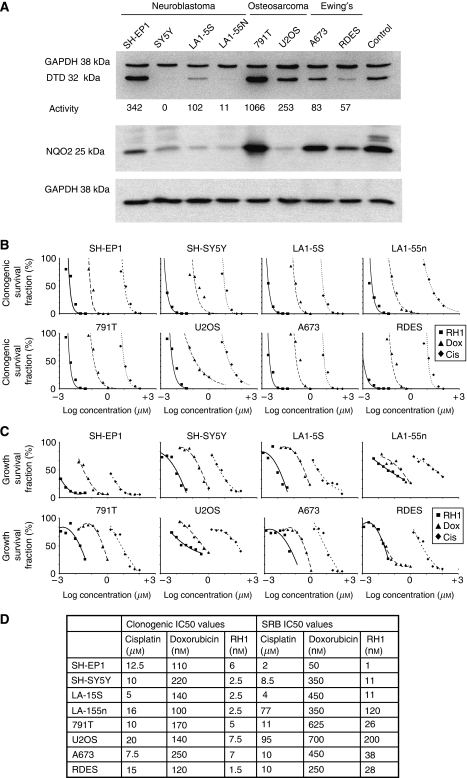
Cytotoxicity of RH1 against paediatric tumour cell lines *in vitro*. (**A**) Immunoblot showing variation in expression of DTD in paediatric tumour cell lines (top), activity of DTD is shown beneath, expression of NQ02 in the same cell lines (bottom). GAPDH is shown as a loading control. (**B**) Clonogenic response of paediatric tumour cell lines to RH1 in comparison with cisplatin and doxorubicin. (**C**) SRB response of paediatric tumour cell lines to RH1 in comparison with cisplatin and doxorubicin. (**D**) Comparison of IC_50_ values in paediatric tumour cell lines for RH1, cisplatin and doxorubicin for clonogenic and SRB assays.

We found a range of expression and activity of DTD in tumour cell lines derived from different paediatric tumours. Of the cell lines examined, DTD was expressed at highest level in the osteosarcoma cell line 791T, and at relatively high level in the U2OS osteosarcoma line (Figure 1A), correlating with their DTD activity. Diphtheria toxin-diaphorase expression and activity in the two Ewing's sarcoma cell lines was lower, but still detectable. In the neuroblastoma cell lines, DTD expression and activity correlated with the type of cell line; S-type neuroblastoma cells had elevated expression and activity compared with their N-type counterparts, both within the MYCN non-amplified, p53 wild type expressing SH-EP1 and SH-SY5Y pair, and within the MYCN amplified, p53 deleted LA1-5S and LA1-55n pair. In the N-type neuroblastoma cell lines, DTD protein expression and activity were very low, and in SH-SY5Y cells, non-existent. Of six rhabdomyosarcoma cell lines three, RD, HX170, and T91-95, expressed detectable DTD, whereas three, SCMC, RMS, and RH18 did not (data not shown). We also evaluated protein levels of the one-electron reductase NQ02, recently suggested to be important for the activation of RH1 in leukaemia and lymphoma cell lines with low levels of DTD (Yan *et al*, 2008). NRH:quinine oxidoreductase 2 was detectable in all of the cell lines examined, although its level varied considerably across the panel. Expression was highest in 791T osteosarcoma cells, which also had the highest level of DTD expression and activity. Importantly, NQ02 expression was detectable in cell lines in which DTD expression and activity was either absent (SH-SY5Y) or very low (LA155n), suggesting that NQ02 may have a role in the activation of RH1 in these cell lines. None of these paediatric tumour cell lines contained the inactivating single-nucleotide polymorphism at position 609 (NQ01^*^2) (Traver *et al*, 1992) that results in loss of DTD activity (data not shown).

### RH1 showed nanomolar potency in inhibiting clonogenicity across the paediatric tumour cell line panel

The efficacy of RH1 at inhibiting colony formation was assessed across the paediatric tumour cell line panel. RH1 potency was superior to the DNA damaging agent cisplatin, and the topoisomerase inhibitor and DNA intercalating agent doxorubicin, both of which are in widespread clinical use against childhood cancer. RH1 was the most potent of the three agents examined, with 1000-fold greater potency than cisplatin, and 19- to 88-fold greater potency than doxorubicin (Figure 1B). Clonogenic IC_50_ values for RH1 varied from 1.5–7.5 nM (Figure 1D). There was no correlation between DTD activity or expression level and sensitivity to RH1 in the clonogenic assay. The IC_50_ values for RH1 against SH-SY5Y neuroblastoma cells, which have no detectable DTD expression or activity, was 2.5 nM, less than that for SH-EP1 neuroblastoma cells (6 nM), which have high level DTD activity, and 791T osteosarcoma cells (5 nM) which showed the highest DTD activity. There was also no correlation between sensitivity to cisplatin or doxorubicin and sensitivity to RH1; U2OS osteosarcoma cells and LA-15S neuroblastoma cells had the same IC_50_ values to doxorubicin but varied three-fold in their sensitivity to RH1, and LA-15S and LA-155n neuroblastoma cells, with the same IC_50_ values to RH1 varied three-fold in their sensitivity to cisplatin. Thus, RH1 is a potent inhibitor of clonogenicity in these paediatric tumour cell lines, and potency is independent of DTD expression or activity.

### RH1 inhibited cell population growth in paediatric tumour cell lines

In keeping with the clonogenic assay results, the comparison of RH1 with cisplatin and doxorubicin in SRB assay of cell population growth showed that RH1 was again the most potent agent, with IC_50_ values ranging from 1 nM for SH-EP1 neuroblastoma cells, to 200 nM for U2OS osteosarcoma cells (Figure 1C). RH1 was between 3- and 50-fold more potent in this assay than doxorubicin, and between 475- and 2000-fold more potent than cisplatin (Figure 1D). In the SRB assay, sensitivity to cisplatin correlated well with sensitivity to RH1; the two cell lines that had IC_50_ values for cisplatin that were roughly 10-fold higher than for the other cell lines (LA-15S and U2OS) also had the only IC_50_ values for RH1 above 40 nM. There was no correlation between sensitivity to RH1 and sensitivity to doxorubicin. Although there was no correlation between expression levels or activity of DTD and sensitivity to RH1, there was a correlation within cell line pairs. For example, SH-EP1 neuroblastoma cells, with relatively high DTD activity, had 11-fold greater sensitivity to RH1 than SH-SY5Y neuroblastoma cells, which had no detectable DTD activity. This 10-fold difference in sensitivity to RH1 between the high and low DTD-expressing cell lines was also seen with the LA1-5S/55n neuroblastoma cells (IC_50_ 11 *vs* 120 nM) and the 791T/U2OS osteosarcoma cells (IC_50_ 26 *vs* 200 nM). The only cell line pair in which there was not a 10-fold difference in IC_50_ to RH1 was the Ewing's sarcoma cell lines, A673 and RDES, in these DTD activity was similar (83 *vs* 57 nM min^−1^ mg^−1^). Thus, paediatric tumour cell lines are sensitive to RH1 in an SRB assay of population kinetics, and in this assay, sensitivity to RH1 correlates with sensitivity to cisplatin, and within tumour types, with DTD activity.

### Differences in sensitivity to RH1 were reflected by differences in RH1-induced apoptosis

**Figure 2:**
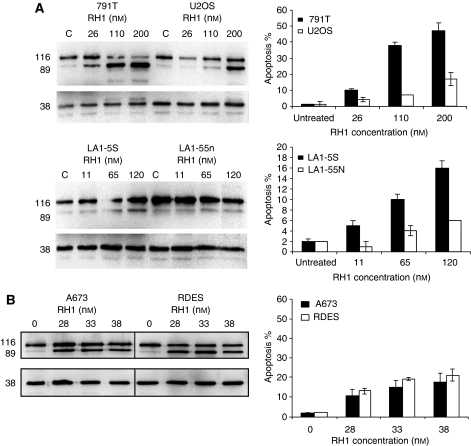
Differential induction of apoptosis by RH1 in paediatric tumour cell lines. (**A**) Differences in SRB IC_50_ values for RH1 between 791T and U2OS osteosarcoma cell lines and LA1-5S and LA1-55n neuroblastoma cell lines are reflected in differences in the induction of apoptosis as measured by nuclear morphology (bar charts) or cleavage of PARP (immunoblots). (**B**) Similar induction of apoptosis between the Ewing's sarcoma cell lines A673 and RDES reflects the lack of difference in SRB IC_50_ values between these two lines, whether measured by nuclear morphology (bar charts) or cleaved PARP (immunoblots).

RH1 has been reported to induce apoptosis in adult breast cancer cell lines. In paediatric tumour cells, RH1 also induced apoptosis as measured 24 h after a 1 h drug exposure by immunoblotting for cleaved PARP and by assessment of classical nuclear apoptotic morphology (Figure 2). The differential sensitivity to RH1 seen in the SRB cell population kinetic assay was mirrored by the levels of RH1-induced apoptosis. Thus, 791T osteosarcoma cells showed greater cleavage of PARP and higher numbers of apoptotic nuclei than did U2OS cells over a concentration range (Figure 2A) correlating with their SRB IC_50_ values of 26 and 200 nM. LA-15S and LA-155n cells also underwent differential induction of apoptosis, again correlating with the differences in their SRB IC_50_ values. With A673 and RDES Ewing's sarcoma cell lines, there was little difference in their IC_50_ values by SRB assay, reflected by similar levels of apoptosis between the two cell lines (Figure 2B).

### RH1 was synergistic with cisplatin and doxorubicin in paediatric tumour cell lines

**Figure 3:**
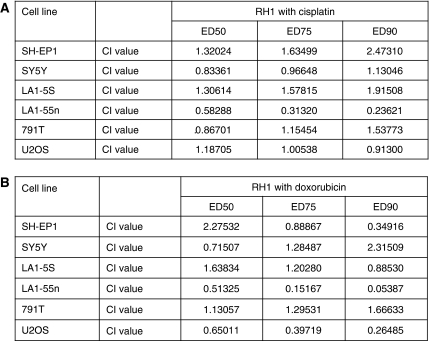
The combination index (CI) values for combinations of RH1 and cytotoxic agents in paediatric tumour cell lines. (**A**) Combination index values for RH1 with cisplatin. (**B**) Combination index values for RH1 with doxorubicin. CI values of 1 indicate additivity, below 1 indicates synergy, and above 1 indicates antagonism. The lower the CI value the greater the degree of synergism.

Earlier studies had shown synergy between RH1 and conventional cytotoxic agents when administered simultaneously (S Danson, personal communication). Thus, the effects of the combination of RH1 with cisplatin or doxorubicin were investigated. The efficacy of fixed ratio combinations of RH1 and either cisplatin or doxorubicin was assessed for all eight cell lines again using the SRB cell population growth assay. Calcusyn software was used to calculate synergy (low combination index (CI) values), additivity (CI values around 1), and antagonism (high CI values). Although strong synergy was seen between RH1 and cisplatin in LA1-55n neuroblastoma cells, at all three concentration levels tested, this was not a constant feature across the panel. In SH-EP1 and LA1-5S neuroblastoma cells RH1 was antagonistic with cisplatin at all three concentration levels (Figure 3A), and in SH-SY5Y neuroblastoma cells and the two osteosarcoma cell lines the drug interaction was additive, with significant antagonism only at ED_90_ concentrations in 791T cells. When RH1 was combined with doxorubicin, evidence of synergy was seen in all cell lines except 791T osteosarcoma cells, in which and in stark contrast, the combination with RH1 was antagonistic at all concentrations (Figure 3B). In LA1-55n neuroblastoma cells and U2OS osteosarcoma cells, there was strong synergy between RH1 and doxorubicin at all concentrations. Interestingly, these are the two cell lines that are least sensitive to RH1 in SRB assay with IC_50_ values of 120 and 200 nM, respectively, compared with a maximum IC_50_ value of 38 nM for the remaining six cell lines.

### RH1 induced apoptosis in paediatric tumour xenografts

**Figure 4:**
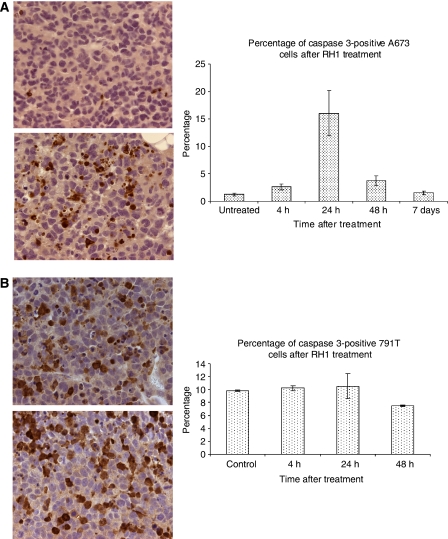
Induction of apoptosis in paediatric tumour xenografts. (**A**) Analysis of cells staining for cleaved caspase 3 in A673 tumour xenografts in nude mice 24 h after a single intraperitoneal dose of RH1. (**B**) Analysis of cells staining for cleaved caspase 3 in 791T xenografts in nude mice 24 h after a single intraperitoneal dose of RH1.

A single i.p. dose of 0.4 mg kg^−1^ RH1 was sufficient to induce apoptosis in A673 Ewing's sarcoma grown as s.c. xenografts in nude mice. Figure 4A shows immunostaining for cleaved caspase 3 (CC3) in A673 xenografts with a clear increase in the proportion of cells expressing CC3 24 h after RH1 dosing (Figure 4B), although this increase was not maintained at later time points. In 791T osteosarcoma xenografts, however, no increase in percentage of cells with CC3 was observed at two time points (24 and 48 h) after RH1 treatment, although in these cells the background level of apoptosis in sham-treated tumours was relatively high and may have masked any RH1-induced effect (Figure 4B).

### RH1 inhibited the growth of paediatric tumour xenografts

**Figure 5:**
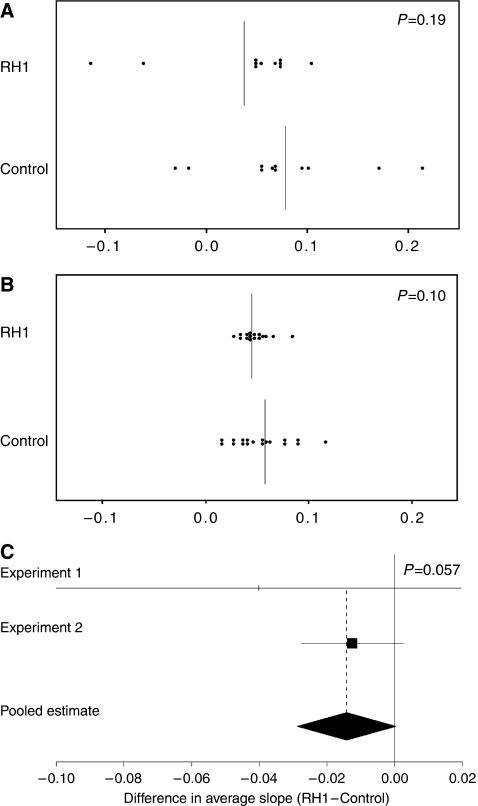
Inhibition of growth of paediatric tumour xenografts by RH1. (**A**) Mean slopes of tumour growth for individual mice bearing A673 xenografts. Vertical lines represent the group mean. (**B**) Mean slopes of tumour growth for individual mice bearing 791T xenografts. Vertical lines represent the group means. (**C**) A forest plot of the comparison between group means for the two experiments, *P*=0.057 for the pooled comparison between RH1 treated and control groups.

A673 Ewing's sarcoma and 791T osteosarcoma xenografts were grown to 200 mm^3^ before random assignment of tumour-bearing mice to treatment with i.p. injection of RH1 (0.4 mg kg^−1^) or DMSO control daily for 5 days. No differences were observed between test and control groups of mice in general health or weight during the course of the experiment, suggesting no general host toxicity of RH1 treatment. Analysis of individual tumour growth rates in a mixed linear effects model allowed the slope of tumour growth to be plotted for each animal (Supplementary Figure 1) and a mean slope for the group to be calculated (Figures 5A and B). The difference in tumour growth in RH1- *vs* vehicle-treated mice across the two tumour types verged on significance (*P*=0.057) (Figure 5C).

## Discussion

This is the first report of the efficacy of RH1 against paediatric tumour cell lines. The data reported here show that paediatric tumour cell lines of various types (neuroblastoma, osteosarcoma, and Ewing's sarcoma) are exquisitely sensitive to this agent *in vitro* where it is more potent than either of the clinically used conventional DNA damaging agents, cisplatin and doxorubicin. RH1 was developed as a substrate for the two-electron reductase DTD, with the aim of selective activation of the agent in tumour tissue. The absolute requirement for and importance of DTD activity for the cytotoxic function of RH1 is controversial. Within modified isogenic pairs of both breast cancer and colon cancer cell lines, RH1 is clearly more effective against the cells expressing higher levels of DTD (Winski *et al*, 1998; Sharp *et al*, 2000; Dehn *et al*, 2005), and indeed such studies have been used to define a threshold level of DTD activity needed for bioreduction of RH1 and a maximum level of DTD activity above which no further activation of RH1 will occur (Winski *et al*, 1998). However, within the NCI 60 cell line panel, there are cell lines without demonstrable DTD activity that remain sensitive to this agent (Tudor *et al*, 2005), which recent data suggest may be due to the activity of NQ02 within these cell lines (Begleiter *et al*, 2007). Diphtheria toxin-diaphorase was variably expressed in the paediatric tumour cell line panel investigated here, which included the neuroblastoma line SH-SY5Y, with no demonstrable DTD expression or activity. As with the NCI 60 panel, no correlation was seen between the efficacy of RH1 in a clonogenic assay and level of DTD activity. Even allowing for the reported narrow window of activity of DTD between threshold and saturation (23–77 nM min^−1^ mg^−1^), two of the neuroblastoma cell lines studied had DTD activity below this threshold (SH-SH5Y 0, LA-155n 11), yet had clonogenic IC_50_ values for RH1 of 2.5 nM, identical to that for LA-15S cells with DTD activity of 103 nM min^−1^ mg^−1^, well above the suggested saturation point for maximum RH1 activation (Winski *et al*, 1998). Thus lack of DTD activity did not lead to RH1 resistance in paediatric tumour cell lines, as with leukaemia cell lines in the NCI60 panel (Tudor *et al*, 2005), and this may be due to the activity of NQ02, which was expressed in all of the paediatric tumour cell lines, at varying levels. In particular, NQ02 expression was detectable in SH-SY5Y cells, which had no DTD expression or activity yet retained sensitivity to RH1. Despite the universal potency of RH1 against paediatric tumour cell lines in clonogenic assay, there were considerable variations in response using the SRB assay, where these differences correlated with the levels of RH1-induced apoptosis. RH1 was a more potent inducer of apoptosis than either cisplatin or doxorubicin. Efficacy of RH1 in SRB assay correlated well with efficacy of cisplatin, and within tumour type the cell line with the higher DTD activity was the more sensitive to RH1, even when, as with the osteosarcoma cell line pair, both cell lines had DTD activity above the postulated upper threshold. It thus seems likely that DTD activity has a role in the cytotoxicity of RH1 in paediatric tumour cell lines *in vitro*, but that other pathways of activation, such as NQ02, are also important. The data also suggest that RH1-induced apoptosis cannot account in full for RH1-mediated reduction in clonogenicity, implicating other cell fates as contributors to the inhibition of colony formation.

Earlier studies have shown rapid onset of apoptosis within 6 h of RH1 treatment, increasing up to 48 h after drug removal (Dehn *et al*, 2005). This is in agreement with the increases in both apoptotic morphology and cleaved PARP that we observed in paediatric tumour cell lines 24 h after RH1 exposure. Differential induction of apoptosis between high and low DTD-expressing cell lines mirrored the differences in sensitivity to RH1 seen in SRB assay. Apoptosis induction showed a better correlation between DTD activity and sensitivity to RH1; 791T osteosarcoma cells have by far the highest DTD activity within the cell line panel and treatment with RH1 induced almost 40% apoptosis at 24 h, approximately 10% apoptosis in U2OS and LA15S cells with intermediate DTD levels, and 4% apoptosis in the DTD low-expressing LA-155n cells (Figure 2A). Although a comparison of apoptosis at a single time point should not be overinterpreted, the data imply that RH1 induction of apoptosis over a 24 h period may be facilitated by the rapid activation of RH1 by DTD to generate proapoptotic cell damage signals. However, as RH1-mediated reduction of clonogenicity is not DTD dependent, cells with low/no DTD presumably accumulate sufficient RH1-induced damage to provoke apoptosis more slowly or RH1 is able to generate alternative cell fate responses that inhibit colony formation.

According to the CI, doxorubicin and RH1 were synergistic in several cell lines, including U2OS osteosarcoma cells, and LA155n and SH-EP1 neuroblastoma cells. Synergy between RH1 and cisplatin was only observed in LA155n neuroblastoma cells. Interestingly, U2OS and LA155n cells, in which the greatest synergy between RH1 and doxorubicin was observed, were the two most resistant cell lines to RH1 in the SRB assay, with IC_50_ values significantly higher than the other lines (LA-155n 120 nM, U2OS 200 nM) and were also relatively resistant to cisplatin. The data suggest that there may be potential benefit in combining RH1 with other cytotoxic agents. However, these *in vitro* methods are merely suggestive of possible synergy *in vivo* and the possibility of greater toxicity when combining several DNA damaging agents needs careful consideration.

In A673 xenografts, RH1 increased the number of apoptotic cells 24 h after a single dose of RH1, in agreement with the kinetics of apoptosis observed *in vitro*. This level of apoptosis was not maintained at later time points, suggesting that repeat dosing of RH1, as used in the *in vivo* experiments, would be required for prolonged effect on tumour growth. RH1 inhibited the growth of both A673 Ewing's sarcoma and 791T osteosarcoma cells grown as xenografts in nude mice. This combination of Ewing's sarcoma and osteosarcoma xenograft data is reflective of the clinical situation where RH1 will be initially evaluated against all relapsed childhood solid tumours.

In conclusion, paediatric tumour cell lines reflecting tumour types with poor prognosis were sensitive to the novel bioreductive agent RH1, regardless of their expression of DTD. In selected tumour types, RH1 was synergistic with doxorubicin *in vitro*. RH1 induced apoptosis in paediatric xenografts grown in mice, and slowed the growth of these xenografts. This data supports further investigation of the usefulness of this agent in the clinic.
