# Supplementary material for: Preclinical efficacy of the bioreductive alkylating agent RH1 against paediatric tumours
Source: Br J Cancer. 2009 Jun 2;101(1):55–63. doi: 10.1038/sj.bjc.6605100 (PMC2713707; doi:10.1038/sj.bjc.6605100)
Supplement: Supplementary Figure 1 [file 6605100x1.ppt]

## Slide 1
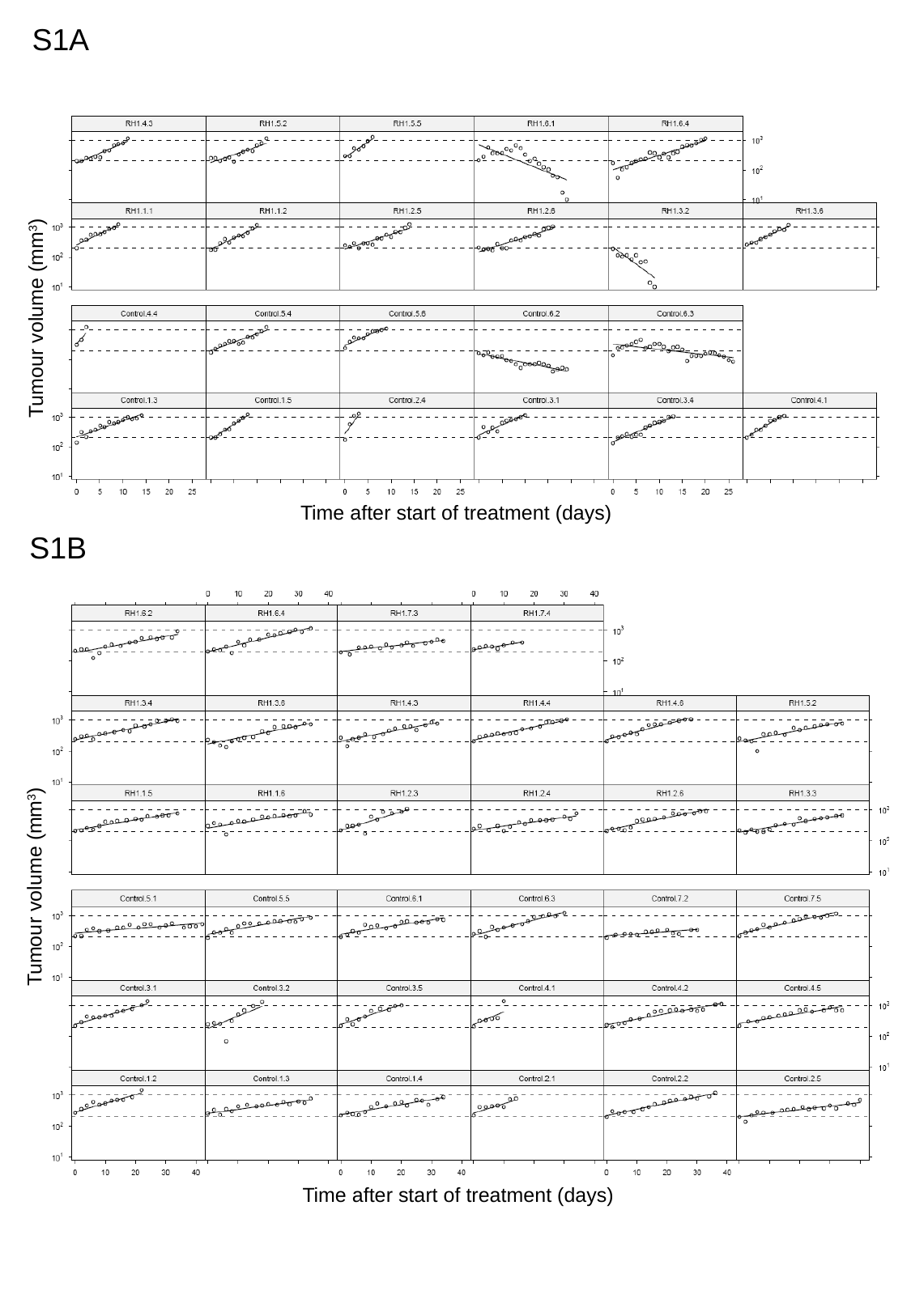

S1A
Tumour volume (mm3)
Time after start of treatment (days)
S1B
Tumour volume (mm3)
Time after start of treatment (days)
