# Supplementary material for: Preclinical efficacy of the bioreductive alkylating agent RH1 against paediatric tumours
Source: Br J Cancer. 2009 Jun 2;101(1):55–63. doi: 10.1038/sj.bjc.6605100 (PMC2713707; doi:10.1038/sj.bjc.6605100)
Supplement: Supplementary Figure Legend [file 6605100x2.doc]

**Supplementary Figure 1.** Plots of log_10_ tumour volume against time for individual mice bearing either A673 xenografts (**A**) or 791T xenografts (**B**), following treatment with RH1 or DMSO.
